# Increasing the Effectiveness of a Physical Activity Smartphone Intervention With Positive Suggestions: Randomized Controlled Trial

**DOI:** 10.2196/32130

**Published:** 2022-03-01

**Authors:** Aleksandrina Skvortsova, Talia Cohen Rodrigues, David de Buisonjé, Tobias Kowatsch, Prabhakaran Santhanam, Dieuwke S Veldhuijzen, Henriët van Middendorp, Andrea Evers

**Affiliations:** 1 Department of Psychology McGill University Montreal, QC Canada; 2 Health, Medical and Neuropsychology Faculty of Social and Behavioural Sciences Leiden Netherlands; 3 Centre for Digital Health Interventions Department of Management, Technology, and Economics ETH Zurich Zurich Switzerland; 4 Centre for Digital Health Interventions Institute of Technology Management University of St.Gallen St.Gallen Switzerland; 5 Department of Psychiatry Leiden University Medical Center Leiden Netherlands; 6 Medical Delta Leiden University, Technical University Delft and Erasmus University Leiden, Delft, Rotterdam Netherlands

**Keywords:** eHealth, mobile health, physical activity, walking, positive suggestions, outcome expectations, mobile phone

## Abstract

**Background:**

eHealth interventions have the potential to increase the physical activity of users. However, their effectiveness varies, and they often have only short-term effects. A possible way of enhancing their effectiveness is to increase the positive outcome expectations of users by giving them positive suggestions regarding the effectiveness of the intervention. It has been shown that when individuals have positive expectations regarding various types of interventions, they tend to benefit from these interventions more.

**Objective:**

The main objective of this web-based study is to investigate whether positive suggestions can change the expectations of participants regarding the effectiveness of a smartphone physical activity intervention and subsequently enhance the number of steps the participants take during the intervention. In addition, we study whether suggestions affect perceived app effectiveness, engagement with the app, self-reported vitality, and fatigue of the participants.

**Methods:**

This study involved a 21-day fully automated physical activity intervention aimed at helping participants to walk more steps. The intervention was delivered via a smartphone-based app that delivered specific tasks to participants (eg, setting activity goals or looking for social support) and recorded their daily step count. Participants were randomized to either a positive suggestions group (69/133, 51.9%) or a control group (64/133, 48.1%). Positive suggestions emphasizing the effectiveness of the intervention were implemented in a web-based flyer sent to the participants before the intervention. Suggestions were repeated on days 8 and 15 of the intervention via the app.

**Results:**

Participants significantly increased their daily step count from baseline compared with 21 days of the intervention (*t*_107_=−8.62; *P*<.001) regardless of the suggestions. Participants in the positive suggestions group had more positive expectations regarding the app (B=−1.61, SE 0.47; *P*<.001) and higher expected engagement with the app (B=3.80, SE 0.63; *P*<.001) than the participants in the control group. No effects of suggestions on the step count (B=−22.05, SE 334.90; *P*=.95), perceived effectiveness of the app (B=0.78, SE 0.69; *P*=.26), engagement with the app (B=0.78, SE 0.75; *P*=.29), and vitality (B=0.01, SE 0.11; *P*=.95) were found. Positive suggestions decreased the fatigue of the participants during the 3 weeks of the intervention (B=0.11, SE 0.02; *P*<.001).

**Conclusions:**

Although the suggestions did not affect the number of daily steps, they increased the positive expectations of the participants and decreased their fatigue. These results indicate that adding positive suggestions to eHealth physical activity interventions might be a promising way of influencing subjective but not objective outcomes of interventions. Future research should focus on finding ways of strengthening the suggestions, as they have the potential to boost the effectiveness of eHealth interventions.

**Trial Registration:**

Open Science Framework 10.17605/OSF.IO/CWJES; https://osf.io/cwjes

## Introduction

### Background

eHealth interventions use information and communications technology, such as mobile phones and computers, to improve or enable health care. The widespread availability of smartphones and computers makes it possible to provide eHealth interventions to broad populations without significant financial costs. A large fraction of eHealth interventions focus on behavioral change and implementing healthy lifestyle habits such as an increase in physical activity and diet change [1]. Accumulating literature shows that eHealth apps can increase physical activity in various groups of the population, including adolescents [2], working-age women [3], older people [4], patients with cardiovascular diseases [5], and survivors of cancer [6]. At the same time, multiple meta-analyses and literature reviews have demonstrated that the effectiveness of various eHealth interventions varies and that the effects of these interventions decrease over time [2,4,7,8]. Several characteristics of the interventions have been proposed to increase their effectiveness: user-friendly design, real-time feedback, and health professional involvement [1].

A possible way of increasing the effectiveness of eHealth interventions, which has not yet been investigated, is to manipulate the expectations of users regarding the intervention. This can be accomplished by providing users with positive suggestions that emphasize the effectiveness of the intervention. Positive outcome expectations are one of the primary mechanisms of placebo effects. A lot of research has been conducted on the effects of positive suggestions on the outcomes of various interventions [9-11]. For example, it has been demonstrated that optimizing the expectations of patients undergoing bypass surgery leads to lower disability in these patients 6 months after surgery [10]. Placebo effects induced by enhancing positive expectations of patients have also been found in pain, itch, depression, fatigue, and nausea [12-15]. In addition to changing the expectations of users, positive suggestions about the intervention can also increase the perceived credibility of the intervention and adherence of the users to the intervention [9,16].

Although placebo effects induced by positive suggestions have been extensively investigated in various areas of health care, studies that look at the potential of using placebo effects in eHealth remain scarce. A recent study has used several types of positive suggestions to change the expectations of participants regarding a smartphone-delivered placebo intervention aimed at improving mood [17] and has found that expectations about the app and its credibility decreased during the 20 days of the placebo intervention. However, this decrease was less prominent in a combined suggestions condition in which participants were informed about the positive effects of the app before the start of the intervention and provided with positive feedback during the intervention [17]. Although this previous study aimed to increase positive expectations regarding a placebo intervention, no study to date has aimed to increase the effectiveness of an active eHealth intervention by changing the expectations of participants.

### Objectives

This study investigates whether positive suggestions can influence the expectations of participants and increase the effectiveness of a physical activity smartphone intervention. We look at the effects of positive suggestions given before and during the intervention on several outcomes. The primary outcomes are (1) expectations of the participants regarding the effectiveness of the app and (2) the number of steps participants took during the intervention. The secondary outcomes are the perceived effectiveness of the app, engagement with the app, and vitality and fatigue of participants during the intervention.

## Methods

### Ethics Approval

This study was approved by the psychology research ethics committee of Leiden University (2020-09-14-AWMEvers- V2-2625). The study protocol was preregistered on Open Science Framework [18].

### Study Design

A randomized, between-subjects study design was used. Participants were randomly allocated to one of the two conditions: (1) positive suggestions group (intervention with positive suggestions) and (2) control group (intervention without suggestions). A random number generator was used to block randomize participants to their condition with a block size of 6.

The study was web based, and all measurements were performed via the internet. During the intervention, participants did not have direct contact with the researchers; however, in case they had questions, they could get in touch with the research team via email.

### Participants

Healthy participants aged between 18 and 40 years were recruited for this study. Recruitment was conducted across the campus of Leiden University and using social media such as Facebook and WhatsApp groups of students. Inclusion criteria were the ability to sufficiently understand, read, and write English; ability to use smartphones and the internet; being in possession of a smartphone; and being willing to increase physical activity. Participants with medical conditions that could hinder a normal physical activity pattern (eg, joint problems or heart disease) were excluded from this study. Participants received €10 (US $11.43) or 8 study credit points for participating in the study in case they completed the whole intervention, €6 (US $6.86) or 6 study credits in case they completed 2 weeks, and €3 (US $3.43) or 3 credits if they completed the first week of the intervention. The reward was given after the end of the study.

As no research so far has been performed on the effects of positive suggestions on the step count during an eHealth intervention, a study on the effects of positive suggestions on physical performance (weightlifting exercises) was chosen for the sample size calculation [19]. The power calculation, conducted with G*Power 3.1 [20], indicated that to detect a difference between positive suggestions and a control group using analysis of covariance, with an estimated effect size of Cohen *f*=0.47 [19], a critical α level of α=.05, and a power of β=.95, 31 participants per group, that is, 62 participants in total, would be needed. In a similar study by our laboratory, which used the same eHealth intervention in healthy volunteers, the dropout rate from the moment of recruitment until the end of the study was 33.6% (46/140). Therefore, we aimed to recruit 93 participants in this study.

### Procedure

The study was advertised as a study testing a mobile phone physical activity intervention. Participants interested in the study were sent an information letter with the details about the study and were asked to digitally sign an informed consent form on the Qualtrics platform (Qualtrics International Inc). Participants were asked to ensure that they had either Google Fit or Apple Health apps installed on their phones at least 1 week before the start of the intervention. The step count data from these apps from the week preceding the intervention were retrieved by the study app and used as a baseline measurement of the average number of steps taken by the participants.

Participants were sent a download link on Apple’s App Store and Google Play Store 1 week before the start of the intervention. After downloading the app, they were asked to fill in several baseline web-based questionnaires measuring their expectations about the app, vitality and fatigue they experienced during the past 2 weeks, motivation to exercise, anxiety, and expected engagement with the app. At this point, they were also asked to give permission to retrieve their step count from Google Fit or Apple Health and send them push notifications.

**Figure 1:**
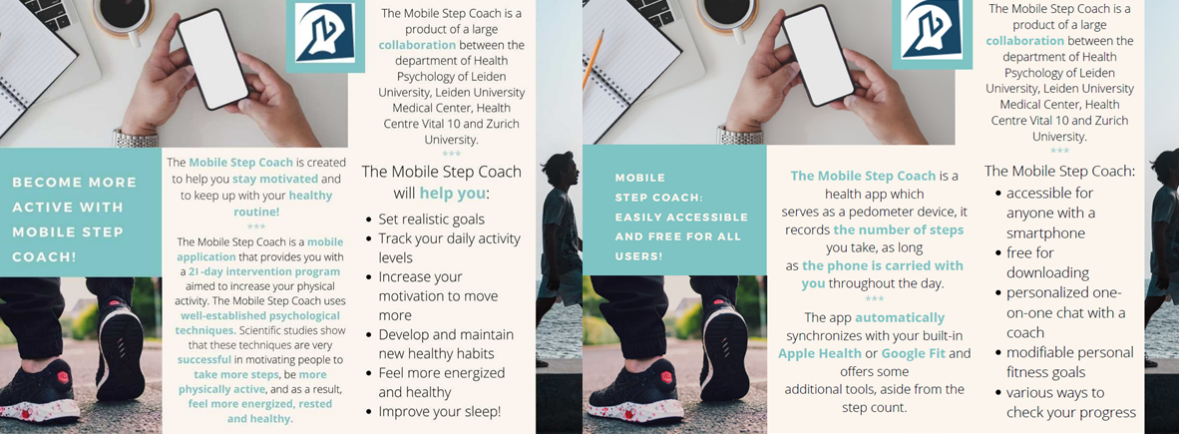
The flyers with positive (left) and control (right) suggestions.

**Figure 2:**
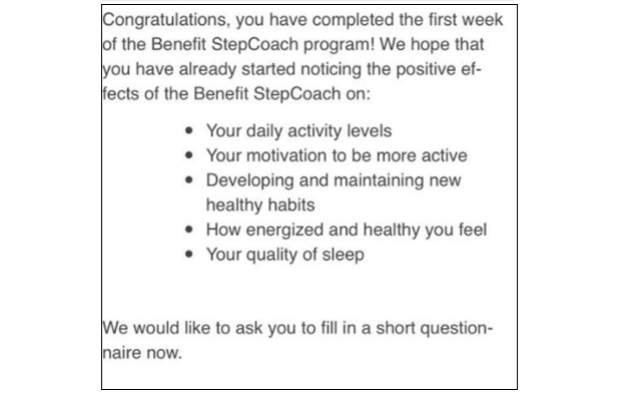
The screenshot of the short suggestions repeated on days 8 and 15 of the intervention.

On day 1 of the intervention, participants were randomly allocated to one of the following intervention conditions: intervention with positive suggestions or control intervention (without suggestions). On the morning of day 1 of the intervention, participants in the positive suggestions group received a web-based flyer with positive suggestions (Figure 1, left). Participants in the control group received a similar flyer describing the technical details of the study app (Figure 1, right). Participants were asked to read the flyers carefully, as the information given on them would be needed during the intervention. After reading the flyers, participants were asked to fill in a questionnaire measuring their expectations regarding the intervention. Every day of the intervention, at 9 AM, participants received a push notification from the study app. In addition, the study app retrieved the step data from Google Fit or Apple Health and tracked the number of steps participants took daily. On days 8 and 15 of the intervention, participants in the positive suggestions group were given brief booster suggestions regarding the intervention (Figure 2), and participants in both groups were asked to fill in short questionnaires about how effective and engaging they found the intervention. After the last day of the intervention, participants were asked to fill in several questionnaires that measured their perceived effectiveness of the intervention, engagement with the app, vitality, and fatigue. The flowchart with the steps of the study is presented in Multimedia Appendix 1.

### Experimental Interventions

#### Study Conditions

##### Positive Suggestions Group

In the positive suggestions group, before the start of the intervention, participants received a leaflet with information regarding the effectiveness of the intervention. It emphasized that the study app has been shown to successfully motivate people to be more physically active and listed various positive effects of using the app. The flyer with positive suggestions is presented in Figure 1 (left).

In addition, the suggestions were implemented in the study app and given to the participants again (booster suggestions) on days 8 and 15 of the intervention (end of the first and second weeks of the intervention). Only 2 very short boosters were given to avoid introducing too much difference in the amount of information that the control and experimental groups had to read. A screenshot of the booster suggestions is presented in Figure 2.

##### Control Group

In the control group, participants received a leaflet with the technical details about the study app. The information was similar in length to the information in the positive suggestions group but did not aim to influence the expectations of participants regarding the effectiveness of the intervention. The control leaflet is presented in Figure 1 (right).

#### The Study App and Intervention

The study app was developed with MobileCoach [21,22], an open-source software platform for smartphone-based and chatbot-delivered behavioral health interventions and ecological momentary assessments. MobileCoach-based interventions [23,24] have been used in various studies for, for example, stress management [25], personality change [26], promotion of health literacy [23], or physical activity [24]. The graphical user interface of the app is similar to WhatsApp or other messaging apps to leverage participants’ expertise in already-existing and well-known communication and interaction paradigms. The app connected to the Apple Health or Google Fit apps on the smartphone of a user and retrieved the step count from these apps every day of the intervention. Moreover, the app had integrated LimeSurvey questionnaires (LimeSurvey GmbH) that allowed the questionnaires to be sent to the users directly through the study app. The MobileCoach-based app of this study aimed to increase the physical activity of its users by increasing their daily step count. The app was tested in other research projects of our laboratory. No bugs or inconsistencies were found in the intervention during the trial. The step data of the participants who finished the intervention were fully available. The step data of participants who stopped with the intervention and did not open the app anymore were not available from the moment they stopped opening the app.

**Figure 3:**
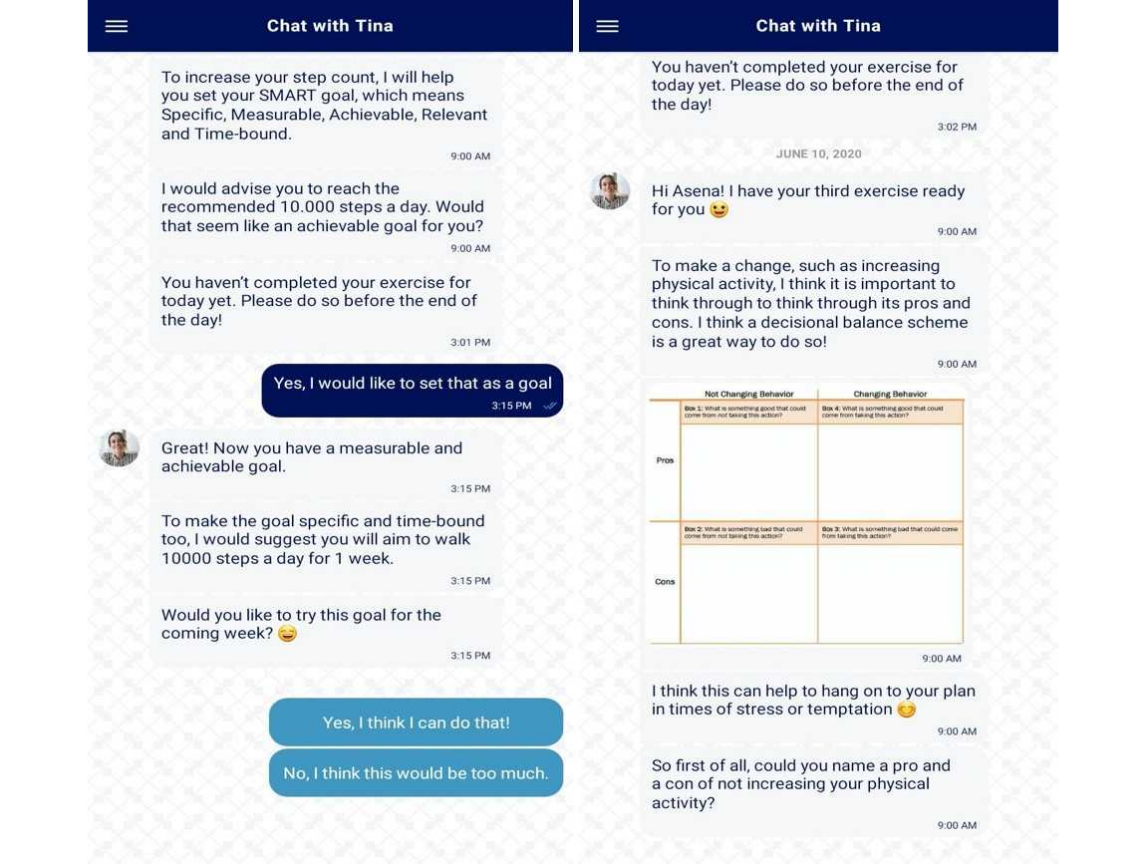
The screenshots of the app.

The study intervention included onboarding and 21 days of active intervention. During the 21 intervention days, every morning, participants received several messages from the chatbot of the app, framed as a mobile step coach. These messages contained short psychological exercises that helped users set reasonable activity goals and think about reasons for participating in the intervention, possible barriers, and ways to overcome these barriers. Similar to other MobileCoach-based interventions [23,24], participants could communicate with the chatbot and type their responses in the app or use predefined answer options. The app used both predefined scripts and predefined answer options for maximum safety and traceability of the intervention progress. The conversational turns with the chatbot included human cues, for example, a picture of the coach, humor, and references to the personal life of the coach, to make the chatbot more human-like [27,28] and trigger a working alliance [23,29,30], which is robustly linked to intervention outcomes [31-33]. A screenshot of the app is presented in Figure 3. As the chatbot was not preprogrammed to converse on topics not related to the intervention, it was not able to reply to the spontaneous questions of participants. If participants needed assistance with the app or had any questions, they could get in touch with the researchers via email.

The intervention was based on the Transtheoretical Model of Health Behavior Change [34]. This model views behavioral change as an upward spiraling process involving progress through five stages: precontemplation, contemplation, preparation, action, and maintenance. Each exercise of the intervention targeted one of the stages. In addition, several behavior change techniques were incorporated into the intervention, such as prompts, cues, information about health consequences, review of goals, and social rewards [35]. An overview of the intervention is presented in Multimedia Appendix 2 (including the stages of the health behavior change targeted by the exercises).

### Measurements and Questionnaires

#### Demographic Characteristics

Participants were asked about their age, gender, nationality, and height and weight. BMI was calculated based on the height and weight of participants using the following formula: weight (kg) divided by height (m^2^).

#### Primary Outcomes

*Steps* were measured by Google Fit or Apple Health on the phone of the participants and retrieved from these apps into the study app. Steps were measured for 1 week before the start of the intervention and for the 21 days of the intervention. The mean number of steps from the week before the start of the intervention was used as a baseline step measure. The number of steps on each day of the intervention was used in the analyses as the main outcome measure.

*Expectations* regarding the effectiveness of the app were measured with six statements created for this study: (1) the app will help me become more physically active; (2) the app will motivate me to increase my step count; (3) the app will help me enjoy moving more; (4) the app will help me develop and maintain healthy habits; (5) using the app will make me feel more energized; and (6) using the app will make me sleep better. Participants were asked to evaluate these statements using a 5-point Likert scale (1=strongly disagree, 2=disagree, 3=neutral, 4=agree, and 5=strongly agree). Expectations were measured twice—at baseline before the flyer was given and after participants read the flyers with positive or control suggestions. The total score of the 6 statements was used in the analysis. The score could range between 6 and 30, with a higher score indicating more positive expectations. The internal consistencies of both baseline (α=.79) and day 1 (α=.80) measures were acceptable and good, respectively.

#### Secondary Outcomes

*Perceived app effectiveness* was measured with six statements created for this study that mirrored the questions about expectations: (1) the app helps me become more physically active; (2) the app motivates me to increase my step count; (3) the app helps me enjoy moving more; (4) the app helps me develop and maintain healthy habits; (5) using the app makes me feel more energized; and (6) using the app makes me sleep better. Participants were asked to evaluate these statements using a 5-point Likert scale (1=strongly disagree, 2=disagree, 3=neutral, 4=agree, and 5=strongly agree). The total score of the 6 statements was used in the analysis. The score could range between 6 and 30, with higher scores indicating more positive expectations. Perceived effectiveness was measured three times: on days 8 and 15 and after the last day of the intervention. The internal consistencies of day 8 (α=.86) and day 15 (α=.87) measures were good and excellent (α=.90), respectively.

*(Expected) Engagement* with the app was measured with the TWente Engagement with EHealth Technologies Scale [36]. Two versions of the questionnaire were used: 1 measuring expected engagement and 1 measuring current engagement. The questionnaire contained 9 statements. The expected engagement questionnaire, measured at baseline and on day 1, asked users about how engaging they thought they would find the app. The engagement questionnaire, measured on days 8 and 15 and at the end of the intervention, asked about how engaging the users found the intervention. Participants were asked to rate the statements using a 5-point Likert scale (1=strongly disagree, 2=disagree, 3=neutral, 4=agree, and 5=strongly agree). The total score could range between 9 and 45, with higher scores on the questionnaire indicating higher engagement with the app. The internal consistency of the baseline measure was questionable (α=.69); however, the day 1 measure was of acceptable consistency (α=.79), and the day 8, day 15, and last day measures had good internal consistency (α=.83, .85, and .83, respectively).

*Vitality* was measured using the Subjective Vitality Scale [37]. The Subjective Vitality Scale comprises 7 statements and measures the subjective feeling of being alive and alert during the past 2 weeks. Participants were asked to rate how true they found the statements on a 7-point Likert scale (1=not at all; 7=very true). The score could range between 7 and 49; higher scores on the composite scale could be interpreted as representing higher self-reported vitality. Vitality was measured at baseline before the start of the intervention and after the last day of the intervention.

*Fatigue* was measured using the Checklist Individual Strength [38]. The Checklist Individual Strength comprises 20 statements that measure four dimensions of fatigue experienced during the past two weeks: fatigue severity, concentration problems, reduced motivation, and activity. Participants were asked to rate how true they found the statements on a 7-point Likert scale (1=yes, that is true; 7=no, that is not true). The score ranged between 20 and 140, and higher scores could be interpreted as higher fatigue. Fatigue was measured at baseline before the start of the intervention and after the last day of the intervention.

*The motivation to exercise* was measured using the Behavioral Regulation in Exercise Questionnaire-2 [39]. The scale comprises 19 items. Participants were asked to indicate how true they found the statements on a 7-point Likert scale (1=not true for me; 7=very true for me). The questionnaire comprises five subscales: amotivation, external regulation, introjected regulation, identified regulation, and intrinsic regulation. Higher scores indicate higher motivation. The motivation to exercise was measured once at the baseline.

*Anxiety* was measured using the Generalized Anxiety Disorder 7-item scale [40]. Participants were asked to indicate how often they had been bothered by a list of problems in the past 2 weeks on a 4-point Likert scale (0=not at all; 3=nearly every day). The score ranged between 0 and 21, with higher scores indicating higher levels of anxiety. Anxiety was measured once at baseline.

### Statistical Analysis

Statistical analyses were performed using RStudio (version 1.1.447; R version 4.0.4). The data and the R code are made available on the web [18]. All tests were performed 2-tailed.

To compare groups on baseline characteristics (age, BMI, steps during the week before the start of the intervention, baseline expectations, fatigue, vitality, baseline expected engagement with the MobileCoach, and motivation to exercise), independent-sample 2-tailed *t* tests or nonparametric Wilcoxon tests (in case of violations of assumptions) were used. To examine whether participants enrolled in the first and second rounds of the recruitment differed on the baseline measurements and the study outcomes (baseline steps, baseline and postintervention vitality and fatigue, steps during the intervention, and postintervention vitality and fatigue), independent-sample *t* tests or nonparametric Wilcoxon tests (in case of violations of assumptions) were used.

To investigate whether the MobileCoach intervention had an effect on the number of steps participants took daily, we compared the mean of daily steps participants took in the week before the intervention with the mean of daily steps during the intervention with a paired-sample *t* test.

The lmer function of the nlme package in R (R Core Team, 2013) was used for the linear mixed effects model analyses to test the main hypotheses of the study. The multilevel structure of the data was defined by *day* (level 1) nested in *participants* (level 2). Parameters were estimated using the full maximum-likelihood procedure. In all models, the intercept was allowed to vary randomly across participants. Random slopes did not improve the fit of the models; therefore, they were removed from the final analysis. The effect sizes (Cohen *d*) of all linear mixed effects models were calculated using the EMAtools package. Cohen *d*=0.2 was interpreted as a small effect size, Cohen *d*=0.5 as a medium effect size, and Cohen *d*=0.8 as a large effect size [41].

To investigate whether the suggestions had an effect on the expectations of the participants regarding the effectiveness of the intervention, we used a linear mixed effects model. The expectations of day 1 (postsuggestions) were the dependent variable, and the independent variables were group and baseline expectations (presuggestions).

To examine whether the suggestions had an effect on the steps taken during the intervention, we used a linear mixed effects model approach. Steps per each of the 21 days of the intervention were used as the dependent variable, and group, baseline steps (to control for the individual baseline differences in walking), and day were the independent variables.

To examine whether the suggestions had an effect on the perceived effectiveness of the intervention, a linear mixed effects model was used with the effectiveness of the intervention as the dependent variable and group and day as independent variables.

To examine whether the suggestions had an effect on the expected engagement with the app, a linear mixed effects model was used with postsuggestions expected engagement as the dependent variable and group and presuggestions expected engagement as independent variables. To investigate whether suggestions had an effect on engagement with the app during the intervention, a linear mixed effects model was used with engagement as the dependent variable and day and group as independent variables.

To examine whether the suggestions had an effect on vitality and fatigue, 2 separate linear mixed effects models were used with vitality or fatigue during the intervention as the dependent variable and group and baseline vitality or fatigue as independent variables.

To examine whether postsuggestion expectations had an effect on the dependent variables, several similar models were created with postsuggestion expectations instead of the group as an independent variable and step count, perceived app effectiveness, (expected) engagement, vitality, and fatigue as dependent variables.

## Results

### Participants

In the first round of the study, 93 participants were recruited between November 2020 and December 2020. As the inclusion criteria were presented in the advertisement, no participants had to be excluded based on not meeting the inclusion criteria. However, the dropout rate was larger than expected, and full data of only 39.8% (37/93) participants were available after the first round of data collection. Therefore, the intervention was performed again in February 2021, with 45 more participants recruited into the study to reach the intended sample size. No follow-up was conducted with the participants who stopped prematurely because of the web-based nature of the study. Therefore, the reasons why the participants dropped out remain unknown.

In total, 138 participants were randomized between 2 groups; 133 (96.4%) participants started the study, and the full data of 79 (57.2%) participants were available. The flowchart of the participants included in each step and dropouts is presented in Multimedia Appendix 3. An overview of the missing data is presented in Multimedia Appendix 4. All available data were included in the analyses, and the number of participants included in each analysis is presented in the results.

### Demographics and Baseline Characteristics

Of the 133 participants, 69 (51.9%) participants (n=55, 80% women; n=13, 19% men; and n=1, 1% other) were assigned to the positive suggestions group and 64 (48.1%; n=56, 88% women and n=8, 13% men) to the control group. The mean age of the sample was 23.3 (SD 6.1) years. An overview of the baseline characteristics across the groups with comparison tests is presented in Table 1. No differences in characteristics were found between any of the groups. No differences were found in the baseline measurements and the outcomes of the study between the participants who took part in the first and second rounds of the recruitment.

**Table 1:** Mean scores of the baseline variables per group and the comparison statistics (N=133).

Variable		Positive suggestions group (n=69)		Control group (n=64)		*t* test (*df*)	Wilcoxon test^a^	*P* value
		Value, mean (SD)	Value, n (%)	Value, mean (SD)	Value, n (%)			
Age (years)		24.00 (6.79)	69 (100)	22.45 (5.26)	64 (100)	—^a^	1939	.22
BMI (kg/m^2^)		23.08 (4.52)	66 (96)	22.71 (3.38)	61 (95)	—	2004	.97
Steps		4060.47 (2271.2)	55 (80)	4172.77 (2576.11)	53 (83)	—	1457	.99
Expectations about app effectiveness		21.29 (2.87)	69 (100)	21.05 (3.12)	64 (100)	—	2171	.87
Expected engagement with the study app		30.06 (2.75)	69 (100)	30.20 (2.86)	64 (100)	0.30 (131)	—	.77
Vitality		4.25 (0.88)	69 (100)	4.28 (0.87)	64 (100)	0.25 (131)	—	.81
Fatigue		30.12 (9.61)	69 (100)	30.98 (8.95)	64 (100)	0.54 (131)	—	.59
Anxiety		5.49 (3.97)	69 (100)	6.05 (4.7)	64 (100)	—	2279	.75
**Motivation to exercise**								
	Amotivation	5.61 (2.49)	69 (100)	5.62 (2.57)	64 (100)	—	2170	.85
	External	6.04 (2.65)	69 (100)	6.23 (3.16)	64 (100)	—	2178	.89
	Introjected	8.54 (3.37)	69 (100)	9.45 (3.27)	64 (100)	—	2508	.18
	Identified	14.46 (3.72)	69 (100)	14.59 (3.82)	64 (100)	—	2255	.83
	Intrinsic	13.94 (4.6)	69 (100)	13.59 (4.36)	64 (100)	—	2256	.83

^a^Wilcoxon test coefficient was used in case a variable was not normally distributed.

### Primary Analyses

#### Expectations

**Figure 4:**
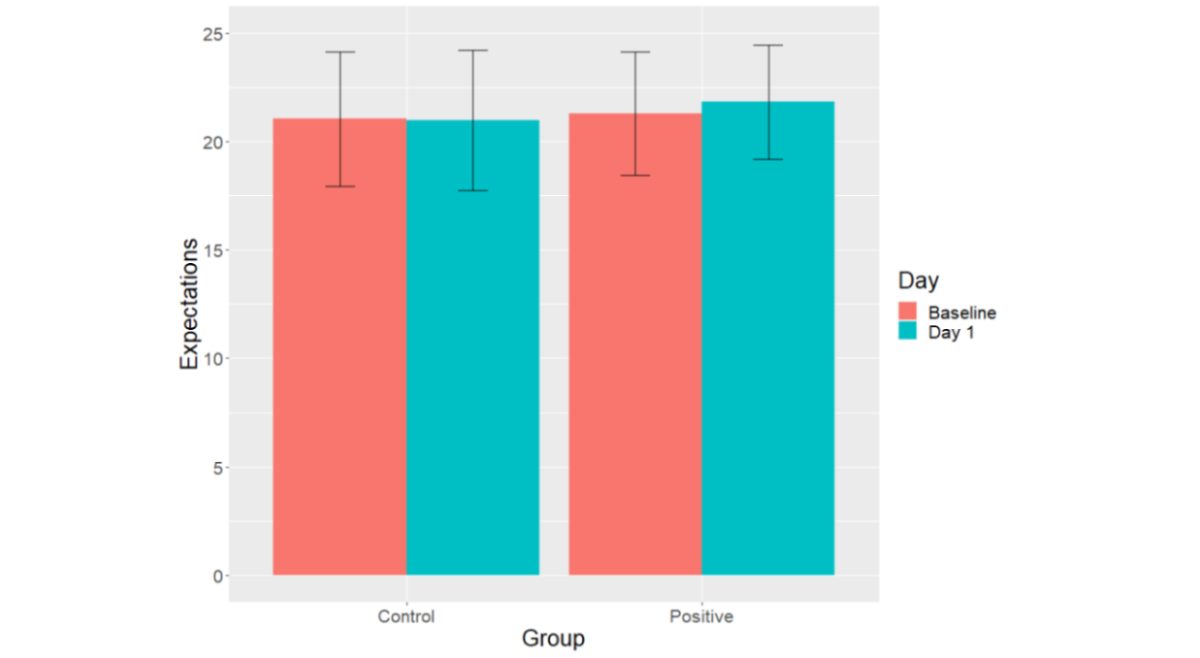
Mean expectations scores per group at baseline and after the flyer with suggestions was sent to participants on day 1. The error bars indicate SDs.

Expectations significantly differed between groups, with a medium effect size (B=−1.61, SE 0.47; *t*_124_=−3.40; *P*<.001; Cohen *d*=0.61; 125/133, 94%) when controlling for the presuggestion expectations: participants in the positive suggestions group (mean 21.82, SD 2.64) expected the app to be more effective than participants in the control group (mean 20.98, SD 3.23). The mean expectations for each group are presented in Figure 4.

#### Steps

Irrespective of the group, participants significantly increased the number of daily steps during the intervention (mean 5689.6, SD 2718.3) compared with the mean number of daily steps during the week before the intervention (mean 4115.5, SD 2414.9; *t*_107_=−8.62; *P*<.001; 108/133, 81.2%).

**Figure 5:**
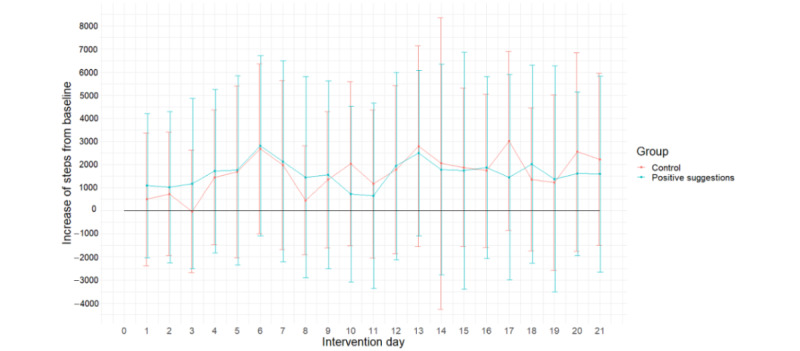
The difference between the mean number of steps taken during the intervention per day and the baseline mean steps per group. The error bars indicate SDs.

The difference between the steps performed on each day of the intervention and the mean number of steps from the baseline week is shown in Figure 5. The multilevel model with the number of steps during the intervention as a dependent variable; baseline steps, day, and group as predictors; and a random intercept (108/133, 81.2%) demonstrated no significant effect of the group on the number of steps (B=−22.05, SE 334.90; *t*_105_=−0.07; *P*=.95; Cohen *d*=0.01). The model showed that the variable day significantly predicted the number of steps: participants in both groups increased the number of steps they took daily during the intervention (B=37.47, SE 12.51; *t*_2009_=2.99; *P*=.003). However, the effect size of the day as a predictor was negligible (Cohen *d*=0.13). Baseline steps also significantly predicted the steps taken during the intervention with a large effect size (B=0.71, SE 0.07; *t*_105_=10.48; *P*<.001; Cohen *d*=1.97): participants who took more steps in the baseline week also took more steps during the intervention.

The model with postsuggestion expectations instead of the group (108/133, 81.2%) demonstrated that expectations did not influence the number of steps taken during the intervention (B=−44.06, SE 59.68; *t*_105_=−0.74; *P*=.46; Cohen *d*=0.14). The effects of the day (B=37.56, SE 12.51; *t*_2009_=3; *P*=.003; Cohen *d*=0.13) and baseline steps (B=0.70, SE 0.07; *t*_105_=10.08; *P*<.001; Cohen *d*=1.97) were also significant in this model.

### Secondary Analyses

#### Perceived App Effectiveness

The reported effectiveness of the app is presented in Multimedia Appendix 5.

The multilevel model with the app effectiveness as a dependent variable, group and day number as predictors, and a random intercept (122/133, 91.7%) demonstrated that the group did not influence the perceived app effectiveness (B=0.78, SE 0.69; *t*_120_=1.12; *P*=.26; Cohen *d*=0.21). The day variable had an effect on the effectiveness: participants reported an increase in the effectiveness of the app during the intervention (B=0.06, SE 0.02; *t*_210_=2.81; *P*=.005; Cohen *d*=0.39).

#### (Expected) Engagement With the App

The multilevel model with group and baseline (presuggestions) expected engagement as predictors and random intercept varying across participants (127/133, 95.5%) demonstrated that the group had a significant effect on the postsuggestions expected engagement (B=3.80, SE 0.63; *t*_124_=6.04; *P*<.001; Cohen *d*=1.09) when controlling for the presuggestions expected engagement: participants in the positive suggestions group (mean 31.62, SD 3.31) expected the app to be more engaging than participants in the control group (mean 30.41, SD 3.26).

Another model with engagement as a dependent variable, and group (122/133, 91.7%) and day number as predictors demonstrated that neither group (B=0.78, SE 0.75; *t*_120_=1.06; *P*=.29; Cohen *d*=0.19) nor day (B=0.042, SE 0.03; *t*_210_=1.60; *P*=.11; Cohen *d*=0.22) had an effect on the reported engagement.

#### Vitality

The multilevel model with vitality during the intervention as a dependent variable, group and baseline vitality as predictors, and a random intercept (117/133, 88%) demonstrated that the group did not have an effect on vitality during the intervention (B=0.01, SE 0.11; *t*_114_=−0.07; *P*=.95; Cohen *d*=0.01). Baseline vitality was positively associated with vitality during the intervention (B=0.20, SE 0.02; *t*_114_=11.11; *P*<.001; Cohen *d*=2.08).

#### Fatigue

The multilevel model with fatigue during the intervention as a dependent variable and group and baseline fatigue as predictors (133/133, 100%) demonstrated that the group significantly predicted fatigue (B=3.09, SE 0.59; *t*_130_=5.22; *P*<.001; Cohen *d*=0.92): participants in the positive suggestions group (mean 23.49, SD 12.47) reported lower fatigue than participants in the control group (mean 24.94, SD 11.62). Baseline fatigue positively predicted fatigue during the intervention (B=0.11, SE 0.02; *t*_130_=6.68; *P*<.001; Cohen *d*=1.17).

#### Postsuggestion Expectations

Postsuggestions expectations significantly influenced the perceived app effectiveness (B=0.62, SE 0.10; *t*_119_=6.13; *P*<.001; Cohen *d*=1.12; 121/133, 91%): the higher expectations participants had about the app on day 1, the higher the app effectiveness they reported during the intervention. Postsuggestion expectations also significantly, and with a large effect size, influenced the vitality of participants (B=0.02, SE 0.004; *t*_114_=5.09; *P*<.001; Cohen *d*=0.95; 117/133, 88%): higher expectations were linked to higher vitality during the intervention. The postsuggestion expectations had no effect on fatigue (B=−0.04, SE 0.02; *t*_124_=−1.79; *P*=.08; Cohen *d*=0.32, 127/133, 95.5%).

## Discussion

### Principal Findings

The aim of this study was to investigate whether it is possible to improve the effectiveness of a physical activity smartphone intervention through positive suggestions. We demonstrated that positive suggestions affected the expectations of participants regarding the effectiveness of the app; however, they were not effective enough to affect the main outcome; that is, daily step count. In addition, participants in the positive suggestions group reported higher expected engagement with the app and lower fatigue during the intervention than the participants in the control group.

The smartphone intervention was effective in helping people increase their physical activity. Participants in both groups walked on average 1500 (SD 2517) steps more during the intervention than before the intervention. Moreover, there was some indication that participants increased their daily steps as the intervention progressed; however, despite the significant effect, the effect size was negligible. This result was expected as the intervention included the phases of the Transtheoretical Model of Health Behavior Change that starts with precontemplation and contemplation about the behavior change and ends with the maintenance of new health behavior [34]. A number of studies demonstrated that physical activity interventions based on the Transtheoretical Model of Health Behavior Change could efficiently increase physical activity in various populations [42-44]. In addition, multiple other smartphone interventions based on different theoretical approaches were shown to increase the physical activity of app users [2,3,8]. Therefore, our results are in line with the literature showing that a psychological smartphone intervention can be successful in helping people increase their physical activity, at least in the short term.

Two outcomes were considered primary in this study: expectations of participants regarding the effectiveness of the intervention and the number of steps taken during the intervention. We hypothesized that positive suggestions would change the expectations of participants, and these increased positive expectations, in turn, would lead to a better outcome of the intervention—an increase in the daily step count. This model is often described in the literature as a working mechanism of placebo effects: positive suggestions induce a change in expectations, and positive expectations lead to better treatment outcomes [16]. The first part of our primary hypothesis was supported by the data: participants in the positive suggestions group indeed reported expecting better effectiveness of the app than the control group. Although the change in the expectations score was quite small, the effect size was shown to be medium. This result is in line with the broad literature on placebo effects: multiple studies demonstrated that positive suggestions given by a health care professional, or an experimenter, can change the expectations of people regarding various types of interventions—in pain [45,46], itch [47,48], nausea [49,50], and many other symptoms [13,15,51].

Despite the effectiveness of the expectancy manipulation, changing the expectations of participants was not enough to influence their daily step count: no difference was found between the groups in their number of steps during the 21 days of the intervention. Moreover, expectations regarding app effectiveness were not predictive of the number of steps participants made, which may explain why an increase in expectations did not affect the step count. These results do not support our main hypothesis and contradict the literature on treatment expectations [52]. The reason for this contradiction can be that our study differs from other studies that used positive suggestions. Most of the research applied positive suggestions focused on symptom-reducing interventions, such as interventions decreasing pain [45,46], itch [53], or nausea [54] or improving mood [55,56]. The intervention that we used in our study aimed to change the behavioral habits of people and required active action from users. Different mechanisms might be involved in interventions that relieve subjective symptoms, such as pain, and interventions that target to change behavior. We can speculate that expecting an overall positive change is not specific enough to improve the outcomes of such interventions and affect their behavior. According to the self-efficacy theory of Bandura [57,58], self-efficacy beliefs have to be very specific to successfully influence behavior, and possibly this specificity was lacking in the suggestions we gave. Generic positive suggestions about an intervention might be more powerful for some subjective outcomes, such as fatigue, which was influenced by positive suggestions in our study. Similar results were found in previous research aimed at influencing food choices by positive suggestions: suggestions influenced subjective food preferences but not actual behavior [59,60]. Our results confirm the results of multiple studies that have demonstrated that fatigue can be decreased by positive suggestions regarding various placebo treatments [19,61].

Another explanation for the failure to confirm the main hypothesis for step count could be the fact that the suggestions given in this study were quite minor. Participants received a flyer with suggestions at the beginning of the study, and the suggestions were briefly repeated on days 8 and 15 of the intervention. It is possible that if the suggestions were more extensive and implemented in each element of the intervention itself, they might have affected the physical activity of the participants.

Finally, the possibility exists that the suggestions did not influence the number of steps but some other parameters that, in turn, could have affected fatigue. The app was not able to measure the pace of walking or the frequency of walking of participants. If positive suggestions affected the walking pace and frequency, these increases in physical activity could have caused the experimental group to experience less fatigue during the intervention.

### Limitations

Several limitations of this study have to be addressed. First, the participants of this study were young and healthy university students. This group is very familiar and comfortable with using mobile phone apps. Therefore, the effects found in this study might not be generalizable to older and less technically experienced populations. It can be that older and less technically experienced people would find it harder to use the app, and therefore, the effects of the app could be smaller in this group of people. At the same time, our main goal was to investigate the effects of positive suggestions on app effectiveness, and there is no particular reason for assuming that computer literacy would influence these effects. Second, several other control groups could have been included in the study to enable us to interpret the results more fully. For example, this study did not include a control group that did not participate in the intervention. This fact makes it impossible to pinpoint whether the intervention was the reason for the increase in physical activity of participants. However, as the main aim was to investigate the effect of positive suggestions, we omitted this experimental condition. In addition, a comparison was made between a group that received a flyer with positive suggestions and a group that received a flyer with technical details. The possibility remains that the fact of presenting users technical versus nontechnical information influenced the study results on top of the presentation of positive versus neutral information. Future research should investigate whether nontechnical neutral information about eHealth apps differently influences their effectiveness. Another limitation of the study is that no follow-up was performed. It remains unknown whether participants would maintain the new habits that they developed during the intervention or the positive effects would disappear quickly after the intervention’s end. Moreover, a selection bias may have been present in this study. It was advertised as a study aimed at increasing physical activity, which might have attracted participants who were already interested in changing their lifestyles. It would be interesting to see how effective the suggestions would be in less motivated or less healthy participants, particularly as a recent meta-analysis of the effects of physical activity eHealth interventions demonstrated that clinical and at-risk groups benefit from such interventions more than healthy volunteers [8]. Another possible limitation of this study is that social desirability might have played a role in the effects we have found. Studies show that similar social desirability patterns occur in interactions with conversational agents, especially when the conversational agent is more human-like [62,63]. The study app used human cues to establish a better working alliance with the participants. However, it could have triggered the participants to give more socially desirable answers on subjective self-report measures.

### Conclusions

In summary, we have demonstrated that short positive suggestions regarding the effectiveness of a mobile health app can affect the expectations of users and decrease their fatigue. Although no effect was found on the main outcome—the step count—these results indicate that eHealth interventions might benefit from adding positive suggestions to the description of the apps. Suggestions are an easy and effective way of giving an extra boost to the effectiveness of eHealth interventions, which does not require extra time or investment. Future research should focus on optimizing such suggestions to achieve actual behavior changes.

## Appendix

Multimedia Appendix 1 The flowchart of the study methods.

Multimedia Appendix 2 Overview of the intervention.

Multimedia Appendix 3 Study flowchart depicting the number of participants in each step of the study.

Multimedia Appendix 4 Overview of the missing data per outcome variable.

Multimedia Appendix 5 Mean perceived effectiveness of the app score per group with SDs.

Multimedia Appendix 6 CONSORT-eHEALTH checklist (V 1.6.1).
