# Supplementary figures and images for: Increasing the Effectiveness of a Physical Activity Smartphone Intervention With Positive Suggestions: Randomized Controlled Trial
Source: J Med Internet Res. 2022 Mar 1;24(3):e32130. doi: 10.2196/32130 (PMC8924786; doi:10.2196/32130)

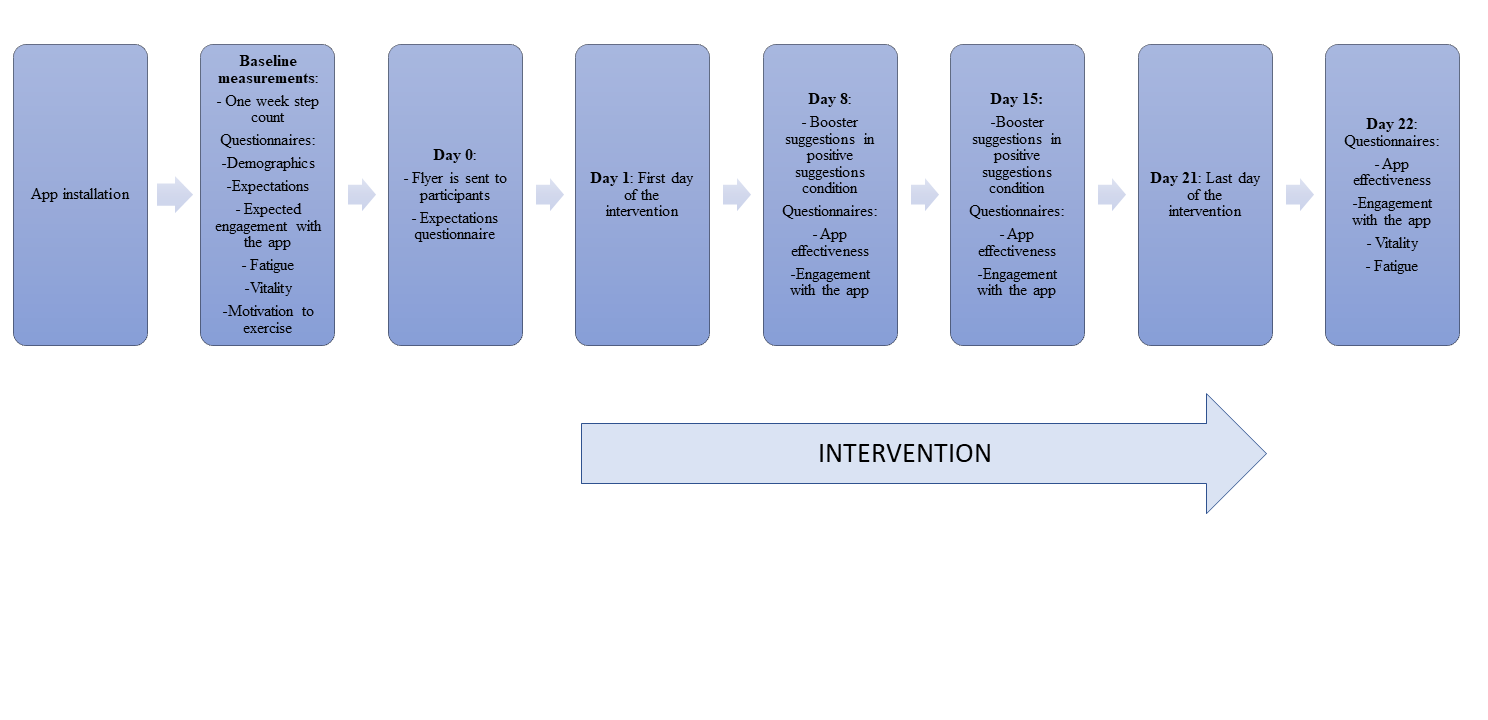

Supplement: Multimedia Appendix 1 [file jmir_v24i3e32130_app1.png]

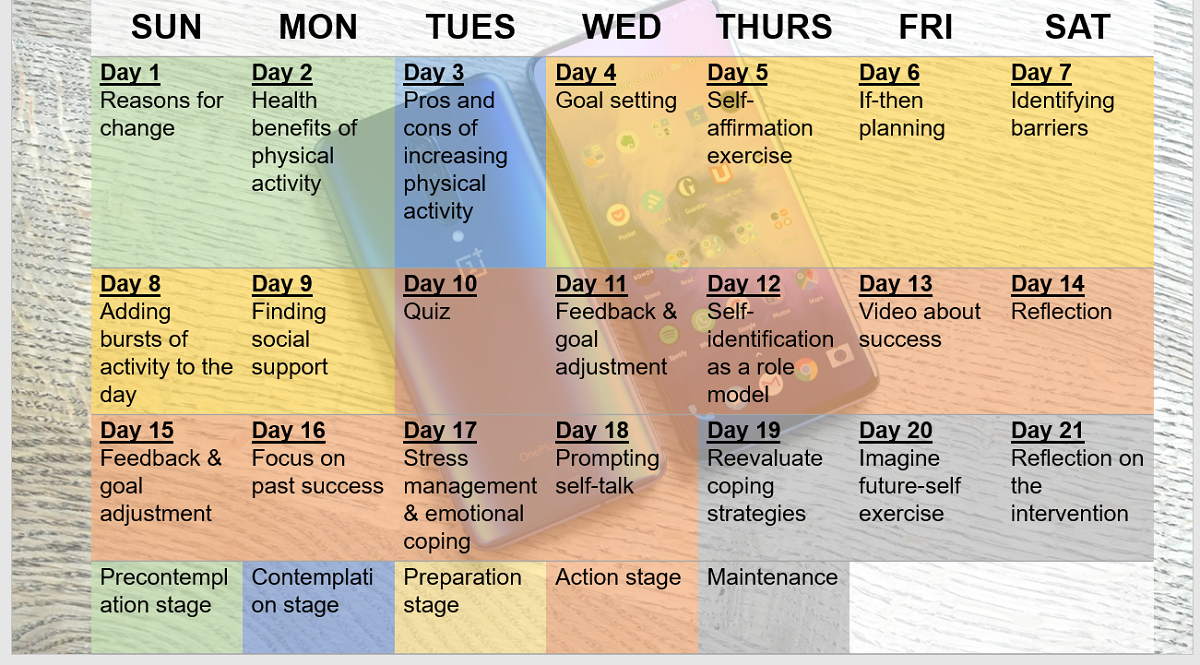

Supplement: Multimedia Appendix 2 [file jmir_v24i3e32130_app2.png]

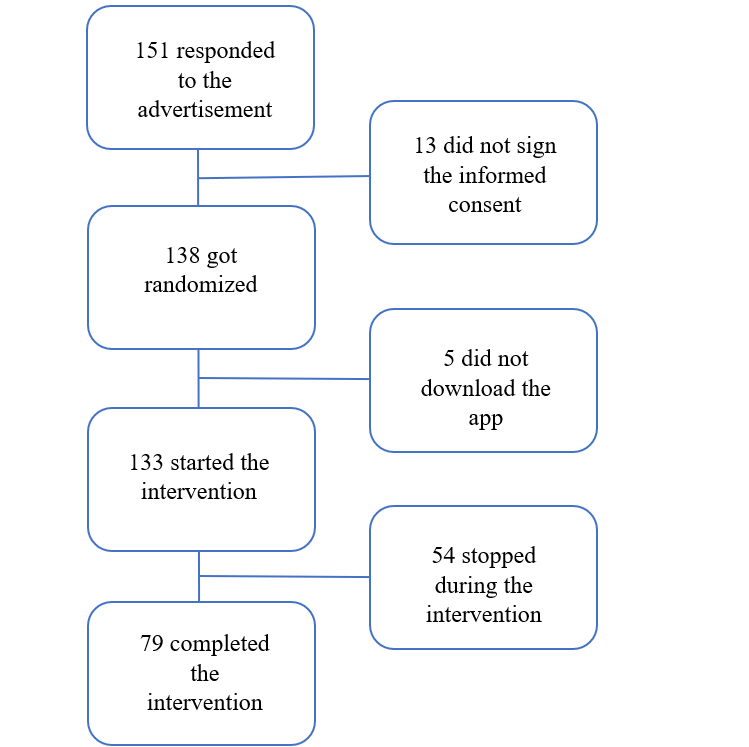

Supplement: Multimedia Appendix 3 [file jmir_v24i3e32130_app3.png]

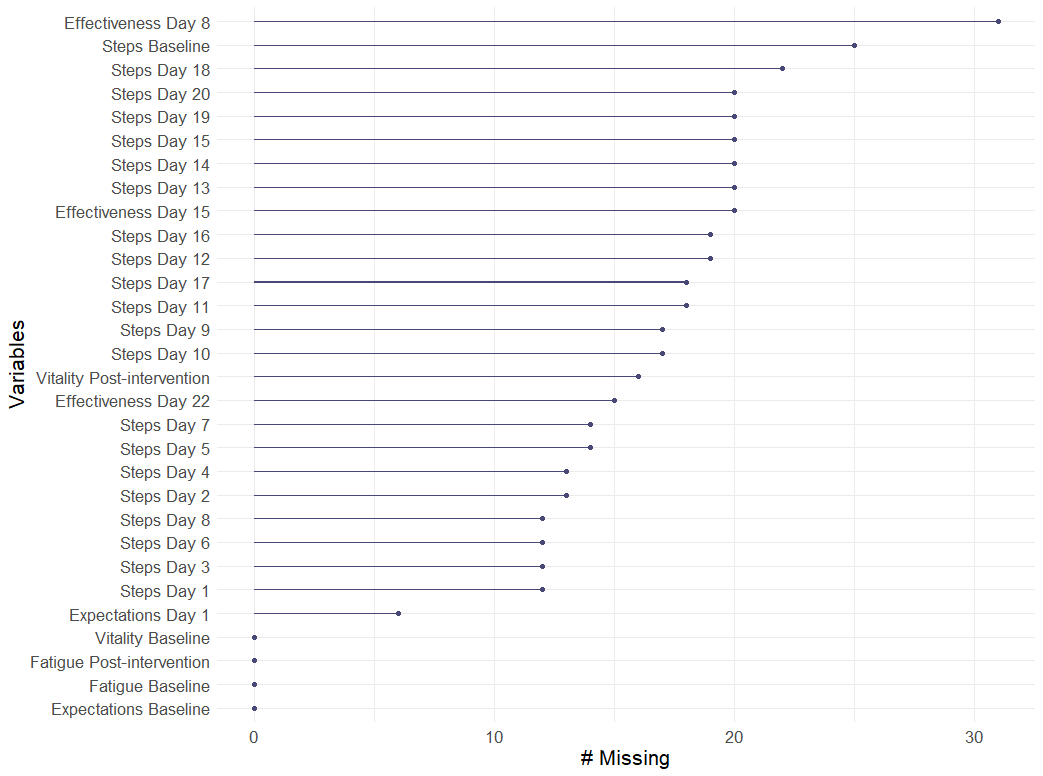

Supplement: Multimedia Appendix 4 [file jmir_v24i3e32130_app4.png]

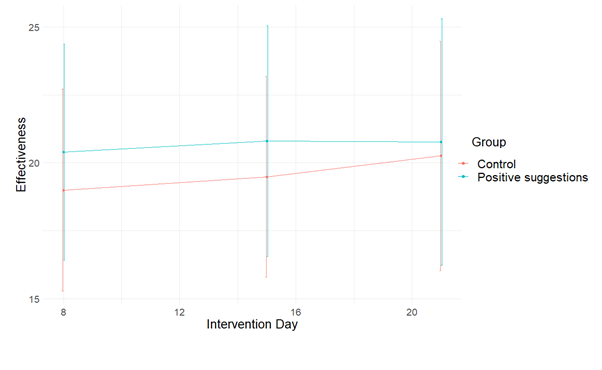

Supplement: Multimedia Appendix 5 [file jmir_v24i3e32130_app5.png]
